# *Nxf7* deficiency impairs social exploration and spatio-cognitive abilities as well as hippocampal synaptic plasticity in mice

**DOI:** 10.3389/fnbeh.2015.00179

**Published:** 2015-07-10

**Authors:** Zsuzsanna Callaerts-Vegh, Tariq Ahmed, Ben Vermaercke, Peter Marynen, Detlef Balschun, Guy Froyen, Rudi D’Hooge

**Affiliations:** ^1^Laboratory of Biological Psychology, University of Leuven, KU LeuvenLeuven, Belgium; ^2^Human Genome Laboratory, University of Leuven and VIB Center for the Biology of DiseaseLeuven, Belgium

**Keywords:** hippocampal synaptic plasticity, nuclear RNA export factor, mouse model, social exploration, spatial learning and memory

## Abstract

Nuclear RNA export factors (NXF) are conserved in all metazoans and are deemed essential for shuttling RNA across the nuclear envelope and other post-transcriptional processes (such as mRNA metabolism, storage and stability). Disruption of human NXF5 has been implicated in intellectual and psychosocial disabilities. In the present report, we use recently described *Nxf7* knockout (KO) mice as an experimental model to analyze in detail the behavioral consequences of clinical NXF5 deficiency. We examined male *Nxf7* KO mice using an extended cognitive and behavioral test battery, and recorded extracellular field potentials in the hippocampal CA1 region. We observed various cognitive and behavioral changes including alterations in social exploration, impaired spatial learning and spatio-cognitive abilities. We also defined a new experimental paradigm to discriminate search strategies in Morris water maze and showed significant differences between *Nxf7* KO and control animals. Furthermore, while we observed no difference in a nose poke suppression in an conditioned emotional response (CER) protocol, *Nxf7* KO mice were impaired in discriminating between differentially reinforced cues in an auditory fear conditioning protocol. This distinct neurocognitive phenotype was accompanied by impaired hippocampal Long-term potentiation (LTP), while long-term depression (LTD) was not affected by *Nxf7* deficiency. Our data demonstrate that disruption of murine Nxf7 leads to behavioral phenotypes that may relate to the intellectual and social deficits in patients with NXF5 deficiency.

## Introduction

Post-transcriptional processes governing transcription, transport and metabolism of neuronal mRNAs are key regulators of dendritic translation, and consequently of synaptic plasticity and cognitive functions. These processes involve a complex system of RNA binding proteins (RBPs) and non-coding RNAs ensuring proper translation activation or suppression with spatial and temporal specificity and precision (Thomas et al., 2014). Mutations in RBPs have been linked to a number of pathologies with associated cognitive dysfunction, such as fragile X mental retardation (e.g., FMRP) and frontotemporal dementia (e.g., TDP-43; Lukong et al., 2008; Tolino et al., 2012).

*Nuclear RNA Export Factor* (NXF) proteins interact with RBPs (Braun et al., 1999; Kang et al., 1999; Bachi et al., 2000), and hence play an essential role in post-transcriptional processes such as shuttling mRNA across the nuclear envelope (Izaurralde, 2002a,b, 2004), cytoplasmic mRNA trafficking (Takano et al., 2007), and mRNA stabilization (Zhang et al., 2007; Katahira et al., 2008). In the human genome, four functional NXF family members have been described (*NXF1*, *NXF2*, *NXF3* and *NXF5*) of which NXF2, NXF3 and NXF5 are clustered on Xq22.1 (Herold et al., 2000; Jun et al., 2001). NXF3 and NXF5 proteins are present in the cytoplasm (Herold et al., 2000; Jun et al., 2001), but *NXF5* appears to be the only one involved in brain development (Jun et al., 2001). *NXF5* was shown to bind non-specifically to RNA, but failed to display RNA nuclear export activity like other NXF proteins (Bachi et al., 2000; Yang et al., 2001).

Several studies in intellectually impaired individuals implicated *NXF5* deficiency, but actual causative information is lacking so far (Jun et al., 2001; Froyen et al., 2007; Grillo et al., 2010). Jun et al. (2001) described a middle-aged man with probable *NXF5* deficiency-related intellectual disability, who displayed relatively unspecific, but severe cognitive impairment, complete lack of speech as well as very limited social and communicative abilities. Grillo et al. (2010) described a female patient with 1.1 Mb deletion of chromosome Xq22.1 containing part of the NXF cluster displaying severe mental retardation, autism, micro-brachycephaly and muscle and bone dystrophies. To investigate the importance of NXF5 in brain functioning more directly, we created a mouse model for this disorder. Based on detailed morphological, histological, and molecular analysis, we recently argued that *Nxf7* is the functional analog of *NXF5*, and that *Nxf7* knockout (KO) mice may therefore represent the most valid model for human *NXF5* deficiency (Vanmarsenille et al., 2013).

In the present study, we investigated various aspects of behavioral and cognitive performance in *Nxf7*-deficient mice as well as hippocampal synaptic plasticity as the cellular correlate of complex learning abilities. We included these particular tests in reference to the intellectual and social defects in *NXF5*-deficient individuals. We assessed emotionality and social exploration, and spatio-cognitive abilities in these mice. Long-term potentiation (LTP) and long-term depression (LTD) of synaptic transmission were analyzed in the hippocampal CA1 region. These synaptic phenomena are widely regarded as cellular correlates of complex information storage in the brain (Huber et al., 2002; Denayer et al., 2008; Zhang et al., 2009; Balschun et al., 2010; Callaerts-Vegh et al., 2012; Lo et al., 2014).

## Materials and Methods

### Animal Colony and Behavioral Test Schedule

*Nxf7*-deficient mice (KO) were generated via targeted deletion of *Nxf7* exon 3 as described in Vanmarsenille et al. (2013). Briefly, homologous recombination of exon 3 in 129Sv embryonic stem cells (ES) cells, and subsequent crosses of chimeric offspring with C57Bl/6 mouse produced male knock-in animals. Deletion of exon 3 was then achieved by mating a knock-in male with PGK-Cre-expressing females. For genotyping, PCR analysis of tail biopsies was performed as described before Vanmarsenille et al. (2013). Standard breeding procedures included crossing heterozygous (HTZ) *Nxf7* females with C57Bl/6 wildtype (WT) males, which resulted in male NXF7 KO offspring. Animals used in behavior had been backcrossed to C57Bl/6 for over 20 generations.

*Nxf7* KO males and their WT littermates were compared using an expanded test battery for motor, emotional, and cognitive performance (Table 1). Behavioral testing started at 8–10 weeks of age. Animals were kept in standard animal cages under conventional laboratory conditions (12 h/12 h light-dark cycle, 22°C) with ad libitum access to food and water (unless stated otherwise). All experiments were conducted during the light phase of their activity cycle. All protocols have been reviewed and approved by the animal experiments committee of the University of Leuven (Belgium), and were carried out in accordance with the European Community Council Directive (86/609/EEC).

**Table 1:** **Overview of behavioral tests, number of animals tested and summary of behavioral observations**.

Protocol	Batch	WT	*Nxf7* KO	*Effect of Nxf7 deficiency*
Spontaneous activity	1	20	22	*Hypolocomotion night time*
Grip	1	20	22	Same as WT
Rotarod	1	20	22	Same as WT
Open field exploration	1	20	22	Same as WT
Social exploration	1	20	22	*Increased center exploration*
Elevated plus maze	1	20	22	Same as WT
Passive avoidance	1	20	22	Same as WT
Morris water maze	1	20	22	*Decreased spatial strategies*
Conditioned emotional responding	1	20	22	Same as WT
Ambiguous cue conditioning	2	18	20	*Impaired auditory cue discrimination*

### General Neuromotor Assessment

Cage activity and neuromotor coordination were assessed by various tests that we described and used previously: 23-h spontaneous cage activity, motor coordination/balance on the accelerating rotarod, and grip strength measurement (D’Hooge et al., 2005). Briefly, 23-h cage activity was recorded using a lab-built activity logger connected to three IR photo beams. Mice were placed individually in 20 × 30 cm^2^ transparent cages located between the photo beams. Over a period of 23 h, activity was recorded as number of beam crossings. To examine motor coordination, mice were first trained on a rotating rod at constant speed (4 rpm, 2 min), before starting four test trials (inter-trial interval, 10 min). During these test trials, the animals had to balance on a rotating rod that accelerated from 4 to 40 rpm in 5 min, and time until they dropped from the rod was recorded (up to the 5-min cut-off). Grip strength was measured using a device consisting of a T-shaped bar connected to a digital dynamometer (Ugo Basile, Comerio, Italy). Mice were placed before the bar, which they usually grabbed spontaneously, and gently pulled backwards until they released the bar (maximal readouts were recorded). Ten such measurements were obtained for each animal.

### Exploration and Anxiety Assessment

Three tests were included that were based on spontaneous exploration. The first two, open field and social exploration, used a 50 × 50 cm^2^ square arena (D’Hooge et al., 2005; Callaerts-Vegh et al., 2006). Animals were placed in the dark for 30 min, and then placed individually in the arena for 11 min (1 min habituation and 10 min recording). Exploration was recorded using EthoVision video tracking equipment and software (Noldus, Wageningen, Netherlands). Total path length and corner crossings were included as measures of locomotor/exploratory activity. Entries into the center of the field were recorded as measures of conflict resolution or anxiolysis. For social exploration assessment, the arena contained a 15 cm round cage with two unfamiliar female mice. Again, exploratory activity was tracked for 11 min (1 min habituation and 10 min recording) using *EthoVision* software using predefined area templates. Finally, an elevated plus maze arena was used to assess anxiety-related exploration (Callaerts-Vegh et al., 2006). The arena consisted of a plus-shaped arena with two arms (5 cm wide) closed by side walls, and two arms without walls. Mice were placed at the center of the cross, and were allowed to explore freely for 11 min (1 min habituation and 10 min recording). Exploratory activity in this arena was recorded by five IR beams (four for arm entries, and one for open arm dwell) connected to a computerized activity logger.

### Spatial Learning and Memory, and Spatio-Cognitive Performance

Spatial learning and memory were examined in a standard hidden-platform Morris water maze using acquisition and retention (i.e., long-term spatial memory) protocols. A 150 cm circular pool was filled with water, opacified with non-toxic white paint, and kept at 26°C as previously described (D’Hooge et al., 2005; Callaerts-Vegh et al., 2006). A 15 cm round platform was hidden 1 cm beneath the surface of the water at a fixed position. Mice were trained for 10 days (5 days followed by a 2-day break) with each daily session consisting of four swimming trials (15–30 min intertrial interval) that started randomly from four starting positions. Mice that failed to find the platform within 2 min were guided to the platform, where they remained for 15 s before being returned to their cages. Probe trials were conducted after five and 10 sessions (after the 2-day breaks and before continuation of acquisition training). During such probe trials, the platform was removed from the pool, and the search pattern of the mice was recorded for 100 s. Swimming paths were recorded using *EthoVision* video tracking equipment and software (Noldus, Wageningen, Netherlands). Search strategies during probe trails were analyzed with binary support-vector-machine (SVM) classifiers (Cortes and Vapnik, 1995). Each probe trial was divided into a time episode before the mouse first reached the virtual platform position, and the episode thereafter. Typically, animals that successfully learned the position of the platform, swim directly towards it and use spatial memory-dependent strategies, whereas less proficient mice employ search strategies that are not as cognitively advanced (i.e., non-spatial or repetitive strategies). We classified the strategy of each mouse during the probe trial periods using a 3-class scoring method (Brody and Holtzman, 2006; Callaerts-Vegh et al., 2012; Lo et al., 2013a).

### Appetitive and Aversive Conditioning

Contextual fear conditioning was assessed with a passive avoidance protocol in a two- compartment box with a shock grid (D’Hooge et al., 2005). The box consisted of an illuminated and a dark compartment, separated by a guillotine door. The dark adapted mouse was placed in the illuminated box and latency to enter the dark compartment was measured. If the subject failed to enter the dark compartment within 5 min trial duration, it was gently probed to enter the dark part. Upon entry into the dark compartment, the door was closed and a 2 s foot shock (0.2 mA) was applied. The mouse was then removed from the box and placed in its home cage. Twenty-four hours later the dark-adapted mouse was again placed in the illuminated box and latency to enter the dark compartment was measured as retention of contextual memory.

Fear conditioning to ambiguous tones was studied using a modified protocol described by Tsetsenis et al. (2007). Testing was conducted in a freezing system (Panlab, Barcelona, Spain), containing a test chamber (26 cm wide × 26 cm long × 27 cm high) with a grid floor placed on top of a force transducer platform to record movements. Test chamber and startle platform were placed inside a ventilated and sound-attenuated cubicle (60 cm wide × 45 cm long × 48 cm high) with a speaker mounted on top of the test chamber. During fear acquisition, animals were presented two auditory stimuli (3 kHz and 8 kHz, 70 dB, 15 s). One stimulus always co-terminated with a mild foot shock (2 s, 0.3 mA; perfect cue, PER: 100% reinforced), while the other tone was either presented alone (no reinforcement) or in compound with the perfect cue and the shock (ambiguous cue, AMB: 50% reinforced; see Figure 4A, schematics of protocol). Each animal received 10 blocks of stimulus presentations (one block consists of (AMB-PER-shock) followed by 1 min inter-stimulus interval followed by AMB) with 1 min intervals between blocks. 48 h later, mice were placed in a novel context (grid floor covered by plastic sheet, changed odors and lighting). Animal movements were detected and recorded for 16 min by the force transducer with a sampling rate of 50 Hz and stored as raw data on a computer. During the first 3 min no stimulus was delivered (baseline freezing in new context), followed by a 5 min presentation of AMB, a 3 min inter-stimulus interval, and a 5 min presentation of PER. 14 days after conditioning, long-term retention was assessed by measuring fear potentiated startle using the same setup. Fear potentiated startle responses resulting from presentation of either the startle stimulus alone (5 kHz, 100–110 dB, 20 ms), or the startle stimulus preceded by the AMB or PER cue were recorded.

Scheduled appetitive conditioning, and acquisition and extinction of fear-induced response suppression were tested in a conditioned emotional response (CER) procedure using automated operant chambers (Coulbourn Instruments, Allentown, PA, USA), essentially as described previously (Callaerts-Vegh et al., 2006). Prior to the training, the animals were placed on a food restriction schedule to keep their body weights at 80–90% of their free-feeding weights. Under increasing reinforcement schedule (FR1-VI30), the animals were gradually shaped to nosepoke for food pellets (Noyes precision pellets, Research Diets, New Brunswick, NJ, USA) in 30 min daily sessions. After obtaining a stable response rate, eight fear acquisition trials with tone-shock presentations were superimposed on the VI-30 s schedule(Callaerts-Vegh et al., 2006), followed by extinction trials. Nosepoke rate during the auditory cues was compared to that during the interstimulus intervals (ISI) by calculating a suppression ratio *(SR) as SR = RRCUE/(RRCUE + RRISI)*; with RRCUE and RRISI representing mean response rates in the presence and absence of the auditory cues, respectively. Thus, a SR of 0.5 indicates complete lack of suppression (equal response rates in the presence and in the absence of the cues), whereas a SR of 0 indicates complete suppression (complete lack of responding in the presence of the cues). Null responders were alloted SR of 0.

### Field Potential Recordings on Hippocampal Slices

The preparation and methods applied were as detailed before Denayer et al. (2008). In brief, experimentally naïve mice of 3–5 months old were killed by cervical dislocation and the right hippocampus was immediately dissected into cold (4°C) artificial cerebrospinal fluid (ACSF) that was saturated with carbogen (95% O_2_ 5% CO_2_). ACSF consisted of (in mM): 124 NaCl, 4.9 KCl, 25.6 NaHCO_3_, 1.2 KH_2_PO_4_, 2.0 CaCl_2_, 2.0 MgCl_2_, 10.0 D-glucose and was adjusted to pH 7.4. Transverse slices (400 μm thick) were prepared from the dorsal area of the hippocampus and transferred into a submerged-type slice chamber where they were maintained at 32°C and continuously perfused with ACSF at a flow-rate of 2.2 ml/min. After 90 min incubation, one slice was arbitrarily selected and a tungsten electrode was placed in CA1 stratum radiatum. For recording of field excitatory postsynaptic potentials (fEPSPs), a glass electrode (filled with ACSF, 3–7 MΩ) was placed in the stratum radiatum. The time course of the fEPSP was measured from their descending slope. After a further hour of incubation, input/output curves were determined, and the stimulation strength was adjusted to maintain a fEPSP slope of 35% of the initial maximum. Paired-pulse ratio (PPR) was investigated by applying two pulses in rapid succession (ISI of 10, 20, 50, 100, 200 and 500 ms) with 120 s between the different measurements. During baseline recording, three single stimuli (0.1 ms pulse width; 10 s interval) were measured every 5 min and averaged.

Synaptic plasticity in form of LTP and LTD were evaluated using previously described protocols (Denayer et al., 2008). Immediately after stimulation, evoked responses were monitored at 1, 4, 7 and 10 min, and then every further 5 min. The magnitude of non-NMDAR mediated post-tetanic potentiation (PTP) in both genotypes was investigated by bath application of the selective N-methyl-D-aspartate receptor (NMDAR) antagonist D-AP5, (D(-)-2-amino-5-phosphopentanoic acid; 50 μM). The drug was applied 10 min prior to delivering a single tetanus (100 Hz-1 s) and 10 min after. All drugs were obtained from Ascent Scientific Ltd. (Bristol, UK). In all cases, experiments on age matched WT and KO mice were interleaved with each other.

### Statistical Analysis

Data are presented as mean ± standard error of the mean (SEM). To analyze behavior readouts, differences between mean values were determined using *t*-test or two-way repeated measures analysis of variance (RM-ANOVA) procedures with Tukey tests for *post hoc* comparison. We used Fisher’s exact test t compare ratios of search strategies between genotypes during the probe trials.

For field potential recordings, group differences between mean values were determined by repeated measures analysis of variance (RM-ANOVA) procedures with Holm-Sidak for *post hoc* comparisons. Intragroup comparisons were performed with Wilcoxon matched-pairs signed-rank test. Student *t*-tests were used for comparisons of PP Rs. All statistical tests were performed at the α level of significance at 0.05.

## Results

### *Nxf7* KO Mice are Hypoactive

**Figure 1:**
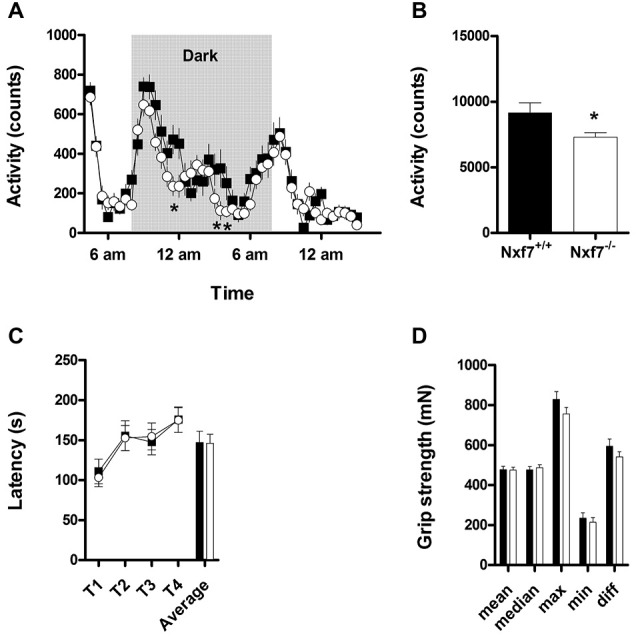
**Activity and neuromotor assessment in wildtype (WT) and *Nxf7* knockout (KO) mice**. Overall 23 h activity was not different between the two genotypes **(A)**, but during the dark period, *Nxf7* KO (open symbols, *N* = 21) were significantly less active than WT mice (black symbols, *N* = 20) **(B)**. Balance on the rotarod **(C)** and grip strength **(D)** was not different between the two genotypes. Data are expressed as means ± SEM. *p < 0.05 *Nxf7* KO vs. *WT* (RM-ANOVA, *post hoc* comparison).

Overall spontaneous cage activity over 23 h was not different between *Nxf7* KO and WT mice (Figure 1A). However, during the dark period, *Nxf7* KO mice displayed a significantly decreased cage activity compared to WT (factor *genotype*: *F*_1,983_ = 5.07, *P* < 0.05 and interaction of factor *genotype* × *time F*_23,983_ = 1.567, *P* < 0.05, Figure 1B). Balancing on the rotating rod and grip strength was similar in *Nxf7* KO and WT mice (Figures 1C,D), indicating a lack of major motor impairment.

### *Nxf7* KO Mice Show Increased Social, but Normal Emotional Exploration

**Figure 2:**
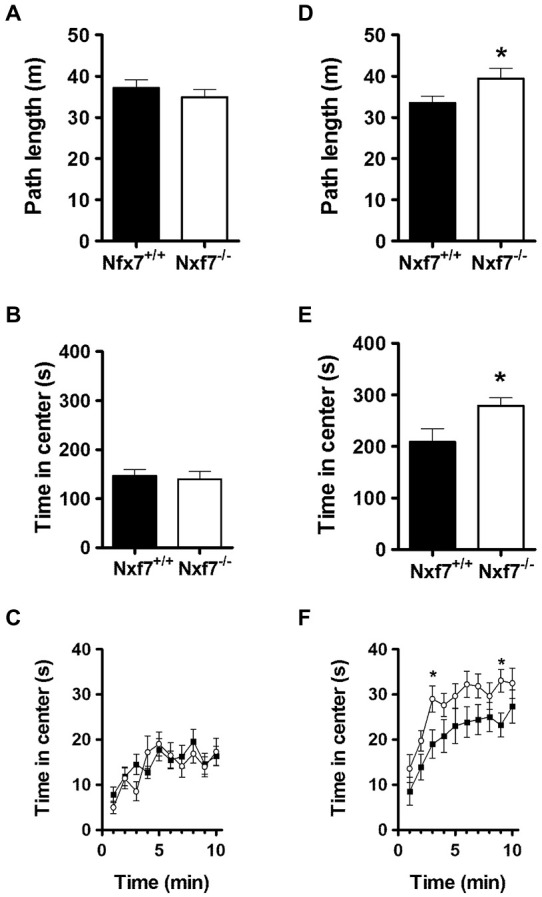
**Exploratory activity in the open field and during social exploration**. In the open field, *Nxf7* KO (open symbols) covered the same distance **(A)**, and spent the same amount of time in the center **(B,C)**, as their WT littermates (black symbols). When two female mice were placed in the center of the open field, male *Nxf7* KO (open symbols) were more active **(D)**, and spent more time in the center **(E)**, and approached the two female mice sooner **(F)** than their WT littermates. Data are presented as means ± SEMs. *p < 0.05 *Nxf7* KO vs. WT.

In open field exploration, no genotype differences were found in path length (Figure 2A), emotionality-related exploration patterns such as path length in the center (11.0 ± 1.0 m and 9.8 ± 1.0 m, for WT and *Nxf7* KO, respectively), thigmotaxis (396 ± 15 s and 416 ± 19 s, for WT and *Nxf7* KO, respectively), or time spent in center (Figures 2B,C).

In social exploration, however, total path length was significantly increased in *Nxf7* KO mice (*t*_38_ = 2.33, *P* < 0.05; Figure 2D). Similarly, run speed was also increased in *Nxf7* KO mice (5.58 ± 0.3 cm/s and 6.57 ± 0.4 cm/s for WT and KO mice, respectively; *t*_38_ = 2.03, *P* < 0.05). This increase in activity was directed towards the two unknown female mice placed in the center of the social exploration field, since *Nxf7* KO spent significantly more time in the center area than WT littermates (*t*_38_ = 2.03, *P* < 0.05; Figures 2E,F). The presence of two female unknown mice induced social approach behavior (increased visit to the center when compared to open field, Figure 2C) and it was sooner and more pronounced in Nxf7 KO mice compared to WT (*time F*_9,342_ = 12.91, *P* < 0.001, *genotype*: *F*_1,38_ = 5.43, *P* < 0.03; Figure 2F).

In elevated plus maze, no differences were observed in general (total arm crosses 123 ± 8 and 126 ± 4, for WT and *Nxf7* KO, respectively), or anxiety-related exploration (open arm dwell: 18.0 ± 1.4 and 21.0 ± 1.2% or open arm crosses: 21.0 ± 1.3 and 23.0 ± 1.6% for WT and *Nxf7* KO, respectively).

### *Nxf7* KO Mice are Impaired in Spatio-Cognitive Performance

**Figure 3:**
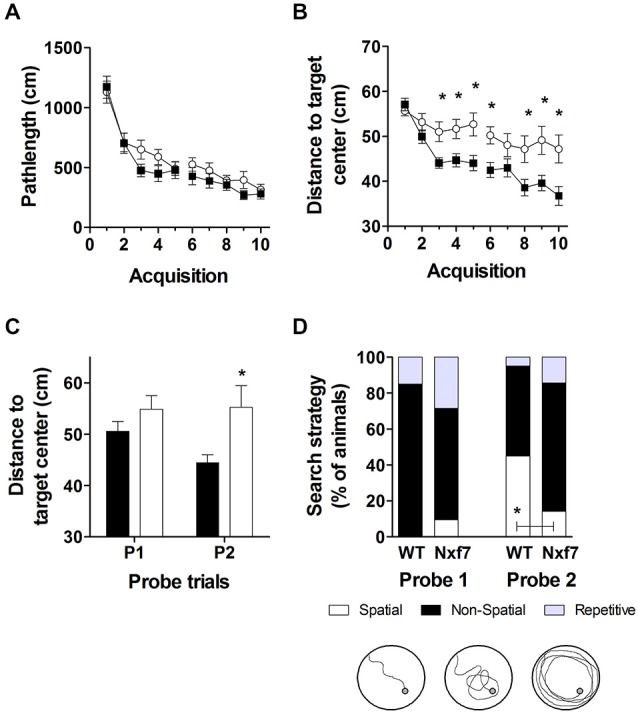
**Spatial learning in the Morris water maze was impaired in *Nxf7* KO (white symbols, *N* = 21) mice compared to WT (black symbols, *N* = 20) mice**. While no difference was found in total path length **(A)**, *Nxf7* KO were less efficient in finding the platform and average distance to the hidden platform was significantly reduced during acquisition **(B)** and during interspersed probe trials **(C)**. Results are presented as means ± SEMs. Search strategy analysis of the probe trials **(D)** indicated that 45% of WT animals employed spatial search strategies in the period after the first virtual platform crossing during the second probe trial In contrast, most *Nxf7* KO mice (71%) relied on non-spatial strategies to search for the platform in the post-period. Tracks are depicting schematic representation of search strategies (for detailed information see Lo et al., 2013a, 2014). Data are presented as ratio. *p < 0.05 comparing to WT. See text for details.

We have previously shown that spatial learning in the Morris water maze was significantly impaired in *Nxf7* KO mice (Vanmarsenille et al., 2013). Further analysis indicated that this lower performance in the mutant group was not caused by differences in path length (Figure 3A, *F*_1,409_ = 2.72, *P* > 0.1), nor in swimming speed (*F*_1,409_ = 1.19, *P* > 0.1, data not shown), but apparently due to lower accuracy in their search for the hidden platform. The cumulative distance between the mouse and the target (platform center), a measure of spatial accuracy, indicated that *Nxf7* KO were less accurate (longer target-mouse distance) than WT mice. RM-ANOVA of distance to the target was significantly different between genotypes (*F*_1,39_ = 8.33, *P* < 0.01), although both groups learned and reduced the distance significantly over trials (effect of *trial F*_9,351_ = 17.43, *P* < 0.001; Figure 3B). In addition, the interaction of *genotype × trial* was significant (*F*_9,351_ = 2.99, *P* < 0.005), consistent with the different learning curves. This difference was also evident during the two probe trials, where the average distance to the target center remained unchanged in *Nxf7* KO mice compared to WT mice (Figure 3C). RM-ANOVA indicated a significant effect for genotype (*F*_1,39_ = 4.58, *P* > 0.05), while the contrasts between probe trials (*F*_1,39_ = 2.44) or the interaction (*F*_1,39_ = 3.15) were not significant. We used a previously described algorithm to categorize the search strategies employed during the probe trial (Callaerts-Vegh et al., 2012; Lo et al., 2013b, 2014). Mice use several strategies to find a hidden platform. Spatial strategies are the most efficient and rely predominantly on cognitive abilities (Brody and Holtzman, 2006; Lo et al., 2013a). However, when the platform is not found, the mouse either continues searching in the same spot (i.e., using spatial strategies), or it switches to non-spatial strategies. To test what strategies are being used, we divided each probe trial in two episodes: the first episode (pre) is defined by the path the animal swims until it crosses the platform position, followed by the period after this event (post). The different episodes were then scored based on the most prevalent search strategy (i.e., spatial, non-spatial and repetitive solutions). The *first episode* in a probe trial (swim to the platform position) is likely similar to the last acquisition trials. The second (after the first platform crossing), however, could yield additional information about robustness of cognitive searching and search strategy employed. During the first probe trial, we observed that the animals used spatial strategies in the pre episode (see Table 2), but after the crossing (post) non-spatial strategies were mostly employed (Figure 3D). No difference was observed between the genotypes. In contrast, during the second probe trial, we observed that both genotypes employed to a similar extent spatial strategies during the pre-episode (45 and 23% for WT and *Nxf7* KO, respectively; Fisher’s exact test not significant). However, for the post episode, WT animals preferred spatial strategies, while *Nxf7* KO mice relied mostly on non-spatial strategies. Fisher’s exact test comparing spatial vs. other strategies between WT and *Nxf7* KO groups indicated a significant association between strategy and genotype (*P* < 0.05, Figure 3D).

**Table 2:** **Search strategies employed during probe trials**.

			WT	*Nxf7* KO
Probe 1	Pre/post	Spatial (%)	55/0	33/9
		Non-spatial (%)	40/85	48/62
		Repetitive (%)	5/15	19/29

Probe 2	Pre/post	Spatial (%)	45/45	23/14
		Non-spatial (%)	45/50	72/72
		Repetitive (%)	10/5	5/14

### *Nxf7* KO Mice are Impaired in Cue Discrimination, but not in Appetitive or Aversive Conditioning

Passive avoidance was acquired in a single session and was tested 24 h later. No differences during training or testing were observed. Latency to enter the dark compartment during training was 14.4 ± 2 and 12.2 ± 1.3 s for WT and *Nxf7* KO, respectively. Twenty-four hours later both groups hesitated to enter (latency to enter 221 ± 28 and 204 ± 25 s for WT and *Nxf7* KO, respectively), indicating that 24 h after conditioning both groups learned to avoid the dark compartment.

**Figure 4:**
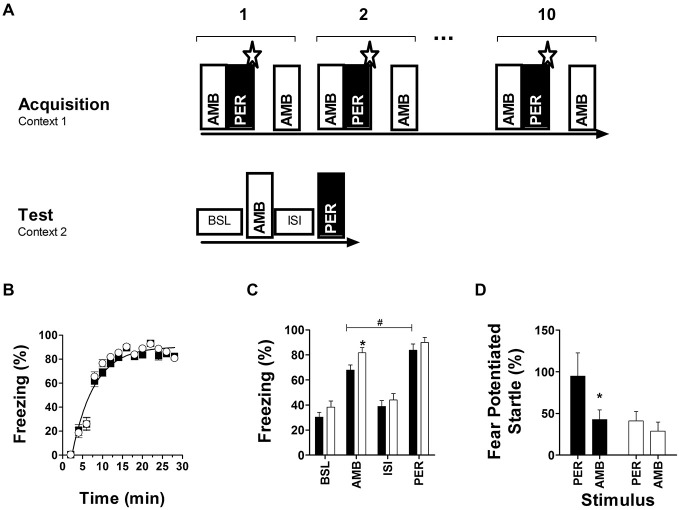
**Fear conditioning to ambiguous auditory cues**. Two auditory cues were presented during acquisition, and tested 48 h later **(A)**. During conditioning, both genotypes developed robust freezing responses to negatively reinforced cues (WT, black symbols, *n* = 18; *Nxf7* KO, white symbols, *n* = 20) **(B)**. Forty-eight hours later memory strength was tested using freezing as a readout during baseline (novel context, no cues), and presentation of either ambiguous (AMB) or perfect (PER) cue. Presentation of the cue induced robust freezing in both genotypes, but *Nxf7* KO mice were generalizing between PER and AMB. (*p < 0.05 *Nxf7* KO vs. WT, ^#^*p* < 0.05 AMB vs. PER) **(C)**. Fourteen days later presentation of PER induced a significant increase in startle response compared to AMB in WT mice. In contrast, *Nxf7* KO displayed a non-differentiating startle response to either cue **(D)** (*p < 0.05, AMB vs. PER). Data are presented as means ± SEM.

Fear conditioning to ambiguous cues indicated that initial fear acquisition was similar in both genotypes (Figure 4B), but that long-term memory accuracy to distinguish between PER and AMB after 48 h (Figure 4C) and 14 days (Figure 4D) was impaired in *Nxf7* KO mice. Two way ANOVA with factors *genotype* and *phase* (BSL, AMB, ISI, PER) for memory retention at 48 h revealed a significant effect of *genotype* (*F*_1,151_ = 6.706, *P* < 0.02), and *phase* (*F*_3,151_ = 63.43, *P* < 0.001). *Post hoc* analysis confirmed that 48 h after conditioning with two cues, WT animals displayed significantly more freezing to the perfect cue (PER) than the ambiguous cue (AMB; *t* = 2.41, *P* < 0.02), indicating that they distinguished between PER and AMB during conditioning. In contrast, *Nxf7* KO mice showed robust cue-induced freezing but showed generalization to both cues (*t* = 1.3; Figure 4C). The robustness of fear memory was demonstrated by measuring fear-potentiated startle to PER or AMB 14 days after conditioning. WT showed a robust startle response to PER, not to AMB, while *Nxf7* KO mice showed no differential response to the two cues (Figure 4D). Two-way ANOVA indicated a significant effect for factor *CS* (*F*_1,55_ = 4.13, *P* < 0.05), *genotype* (*F*_3,55_ = 4.55, *P* < 0.05), without significant interaction. Pairwise comparisons indicated that WT mice had a significantly higher startle response during PER than *Nxf7* KO mice (*t* = 2.39, *P* < 0.05), but not during AMB (*P* > 0.5), and the difference between PER and AMB was significant in the WT group (*t* = 2.17, *P* < 0.05), but not in the *Nxf7* KO group (*P* > 0.5). This effect was not due to differences in startle reactivity to the startle stimuli alone (19.9 ± 4.3 and 20.8 ± 3.8 arbitrary units, *P* > 0.5, for WT and *Nxf7* KO mice, respectively).

**Figure 5:**
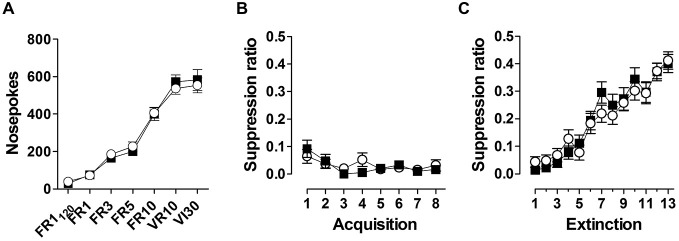
**Animals were trained under increasing reinforcement schedules to obtain food pellets through nose poking (A)**. Reinforced schedules included fixed ratio (FR), variable ratio (VR) and variable interval (VI). Superimposed fear conditioning suppressed instrumental responding robustly **(B)**, which recovered during subsequent extinction trials **(C)**. Data are expressed as means ± SEM. No difference in performance between WT (black symbols) and *Nxf7* KO (white symbols) were observed.

During appetitive conditioning, both genotypes increased nose poking in response to increasing reinforcement schedules, reaching a stable response rate of ~500 nosepokes /30 min (Figure 5A). During superimposed fear acquisition trials, both genotypes show a rapid and stable response suppression (Figure 5B), indicating no difference in fear learning between the two genotypes. Similarly, no difference between genotypes was observed during extinction learning (Figure 5C).

### Long-Term Potentiation but not Long-Term Depression is Impaired in *Nxf7* KO Mice

**Figure 6:**
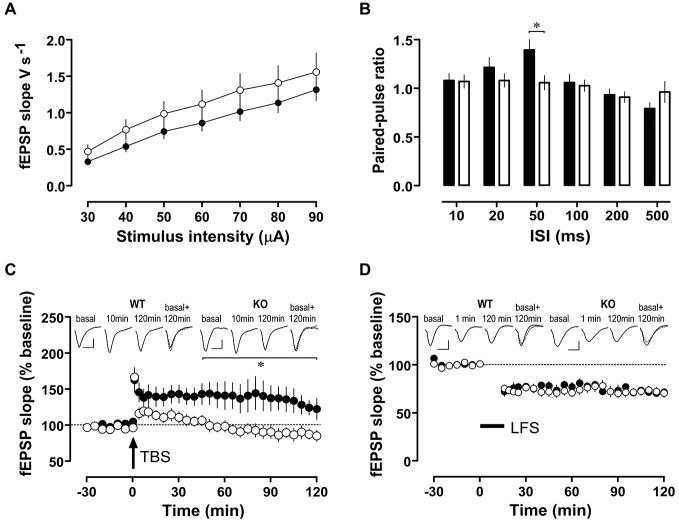
**Synaptic plasticity in area-CA1 of hippocampus from *Nxf7* KO (open symbols) and WT littermates (filled symbols). (A)** Fast synaptic transmission in *Nxf7* KO animals (*n* = 15) was similar to WT (*n* = 17) animals. **(B)** Paired-pulse facilitation was reduced in *Nxf7* KO (*n* = 21) mice at 50 ms ISI compared to WT (*n* = 17; *P < 0.05, Students *t*-test). **(C,D)** Synaptic plasticity in area-CA1 of hippocampus from *Nxf7* KO and WT littermates. **(C)** Long term potentiation induced by theta burst stimulation (TBS) was impaired in *Nxf7* KO mice (*n* = 8) compared to WT animals (*n* = 6). Insets show representative analog traces collected during baseline recording, 10 min after tetanization (to exclude any confounding influence by post-tetanic potentiation) and 120 after tetanization. Traces on the right side display overlaid traces from baseline recording (full line) and 120 min after tetanization (broken line). Calibration bars indicate 0.5 mV and 10 ms, respectively. **(D)** LTD induced by low-frequency stimulation of 900 pulses at 1 Hz was similar in *Nxf7* KO animals (*n* = 12) and in WT (*n* = 8). *p < 0.05, RM-ANOVA. See **(C)** for explanation of insets. Note that for LTD a 1-min-value is shown.

Synaptic plasticity in the form of LTP and LTD are considered cellular correlates of memory processes. While basal excitatory synaptic transmission (input/output curves, Figure 6A) was similar in WT and *Nxf7* KO mice, paired-pulse facilitation, a form of presynaptic short-term plasticity, was reduced in* Nxf7* KO mice at an interpulse interval of 50 ms (*t*_36_ = 2.711, *P* < 0.02; Student’s *t*-test, Figure 6B). When LTP in area-CA1 was examined, potentiation was readily evoked in *Nxf7* KO and WT mice (1 min after TBS: WT 163 ± 17%, *n* = 6; KO 166 ± 8%, *n* = 8, Figure 6C). LTP was maintained for at least 2 h in WT (120 min: 123 ± 15%, *n* = 6), but decayed rapidly in *Nxf7* KO, becoming indistinguishable from baseline already 25 min after tetanization (Wilcoxon matched-pairs signed-rank test). Statistical comparison by RM-ANOVA identified a significant group difference after tetanization (factor *genotype F*_1,363_ = 6.38, *p* < 0.05) and pairwise multiple comparisons (Holm-Sidak) revealed significant differences between *Nxf7* KO and WT mice from 45 min onwards (Figure 6C). To exclude differences in NMDAR-independent PTP, we repeated the same tetanization protocol in the presence of 50 μM D-AP5. In both genotypes, we observed PTP of a similar magnitude as the potentiation without D-AP5 (WT, 159 ± 33%, *n* = 3; KO 166 ± 32%, *n* = 3, data not shown). Low frequency stimulation (LFS) of 900 pulses at 1 Hz resulted in similar stable LTD in both genotypes (1 min after LFS: WT 72 ± 5%, *n* = 8; KO 75 ± 4%, *n* = 12, Figure 6D). To summarize, we observed impaired hippocampal LTP, but intact LTD in *Nxf7* KO, which may be related to their cognitive defects.

## Discussion

In the present study, we modeled the functional consequences of NXF5 deficiency (Jun et al., 2001; Frints et al., 2003; Grillo et al., 2010), using mice deficient for* Nxf7*, the likely murine homolog of human *NXF5* (Vanmarsenille et al., 2013). We examined the effect of genetic deletion of the nuclear export factor *Nfx7* on a variety of behavioral tests and related the observed phenotype to changes in hippocampal synaptic transmission. *Nxf7* KO mice were impaired in employing spatial strategies during acquisition and reference memory tasks in the water maze. We also observed altered social behavior in these mice, whereas motor performance and exploration were similar to WT mice. These behavioral changes coincided with reduced hippocampal LTP, whereas LTD remained unaffected.

Relating phenotypes of patients with loss-of-function mutations to those of disease gene-specific KO mice is often difficult, but *Nxf7* KO mice did present social behavior and learning and long-term memory retention impairments. These deficits are reminiscent of the limited social capabilities and the intellectual disability observed in patients. We previously showed that *Nxf7* male KO mice are healthy, fertile and do not show any obvious constitutional or progressive abnormalities. Furthermore, histological examination of KO brains did not show any alterations (Vanmarsenille et al., 2013). *Nxf7* KO and WT littermates were compared in different functional assays using an extended battery of behavioral tests and synaptic plasticity recordings. No differences were observed in rotarod or grip strength assays indicating that (neuro)motor functions are largely unaffected. Furthermore, *Nxf7* KO were undistinguishable from WT littermates in open field exploration and elevated plus maze, two tests that measure anxiety-related behavior and conflict resolution (Bailey and Crawley, 2009). However, *Nxf7* KO mice showed behavioral alterations in the social exploration protocol. The changes we observed in *Nxf7* mice could be related to loss in social inhibition or to increased sexually motivated approach. Notwithstanding obvious differences in social repertoire between mice and humans, patients with non-functional NXF5 also displayed a wide spectrum of personality disorders, including changes in social interaction (Jun et al., 2001; Frints et al., 2003; Grillo et al., 2010). Changes in social behavior have also been seen in other murine models of human disorder with altered social functions. For example, Pagani et al showed that activation of vasopressin 1b receptors (Avp1b) in hippocampal CA2 region affected social inhibition without changing anxiety-related behaviors (Pagani et al., 2015), whereas polymorphisms in patients were linked to disinhibition (i.e., ADHD-like behavior observed by Zai et al. (2012) and autism spectrum symptoms by Chakrabarti et al. (2009).

In addition to social behavior, we also tested *Nxf7* KO mice in a variety of hippocampus-dependent avoidance and spatial learning tests, as a possible correlate of the cognitive difficulties observed in NXF5 patients. We observed no influence of *Nxf7* deficiency on single-trial passive avoidance learning, a test that relies on simple contextual association (Bartus et al., 1980; Gower and Lamberty, 1993; Webster et al., 2014). Similar to WT littermates, NXf7 mice were able to increase performance in an appetitive conditioning shaping procedure. However, we observed defects in spatial learning and memory performance in the Morris water maze, which has become a prototypical test of hippocampus-dependent learning and memory (Brandeis et al., 1989; D’Hooge and De Deyn, 2001). In particular, the classification of search strategies in cognitive and non-cognitive approaches indicated a preference for non-spatial approaches in Nxf7-deficient mice. Finally, while we observed no difference in fear conditioning, *Nxf7* KO mice were generalizing between two presented cues, a process shown to rely quite selectively on the hippocampus (Tsetsenis et al., 2007; Enkel et al., 2010). The observed defects therefore suggest selective hippocampal dysfunction in *Nxf7*-deficient mice.

Given these behavioral indications of hippocampal dysfunction in *Nxf7*-deficient mice, we investigated hippocampal electrophysiology on brain slices of *Nxf7* KO mice. The electrophysiological recordings revealed changes specifically related to the processes of synaptic plasticity, because basal synaptic transmission was unaltered. Reduced paired-pulse facilitation in *Nxf7* KO mice is furthermore indicative of defects in pre-synaptic processes of short-term plasticity (Zucker and Regehr, 2002). The impairment in hippocampal LTP contrasts with the induction of LTD, which was robust and similar to WT. The role of other NXF-family members in synaptic plasticity (LTP and LTD) has not been investigated, but important functions in synaptic plasticity have been reported for RNA binding/transport proteins such as Staufen and the FMR family (Kao et al., 2010). Down-regulation of Stau1 impaired chemically induced LTP in the CA1 region of organotypic cultures, whereas electrically induced LTP, and LTD induced by group I mGluR activation, were unaffected (Lebeau et al., 2008). Transcriptional silencing of the *FMR1* gene and the concomitant loss of fragile-X mental retardation protein (FMRP) has been shown to have detrimental consequences causing fragile-X syndrome. *Fmr1* KO mice have been characterized extensively, but show quite a different behavioral phenotype from the presently examined *Nxf7* KO mice (Kooy, 2003). In *Fmr1* KO animals, spatial learning and memory were normal, whereas reversal learning was impaired in different tasks. Such mice have unchanged LTP in hippocampal CA1 (Godfraind et al., 1996; Zhang et al., 2009), but group I mGluR-dependent LTD was enhanced (Huber et al., 2002; Zhang et al., 2009). Notably, mice lacking *Fmr2*, a gene associated with mild cognitive impairment in humans, did show an increase in NMDA receptor-dependent and -independent types of CA1-LTP (Gu et al., 2002). Similarly, downregulation of Cyfip1, a direct FMRP interacting protein, had no effect on hippocampal LTP, but increased DHPG-induced LTD (Bozdagi et al., 2012).

We propose that the different behavioral and physiological phenotypes observed upon disruption of Nxf7 and Fmrp could be due to Nxf7 and Fmrp representing distinct subsets of RBPs that regulate different sets of cellular mRNAs. Supportive evidence for this hypothesis comes from a number of different studies. Given the exclusive localization of Nxf7 in cytoplasmic granules (Tan et al., 2005), later defined as translating ribosomes, stress granules and P-bodies (Katahira et al., 2008), and its association with hnRNP A3 and Staufen1, Nxf7 was suggested to play a role in cytoplasmic mRNA transport, translation, degradation and/or storage (Tretyakova et al., 2005; Katahira et al., 2008). Another NXF family member, Nxf2, also controls mRNA dynamics (Takano et al., 2007), but interacts with the neuronal mRNA regulating protein Frmp (Lai et al., 2006; Zhang et al., 2007). Even though Fmrp and Stau1 have been demonstrated in the same RNP granules in *Drosophila* (Barbee et al., 2006; Bolduc et al., 2008) and mouse (Ferrari et al., 2007), they have been observed in separate particles as well (Barbee et al., 2006). Both proteins have been shown to interact with a shared set of RNA transcripts including *Fmr1* and α*CamKII* (Ferrari et al., 2007). Still, 85% of Fmrp-interacting cellular mRNAs were not detected in Stau1-associated transcripts (Furic et al., 2008). Furthermore, *Stau1* KO mice show impaired LTP comparable to our *Nxf7* deficient mice, whereas behavioral and electrophysiological recordings of the *Fmr1* KO mice were very different. Finally two other RBP models did not display cognitive differences in standard hippocampus dependent tests (Stein et al., 2006; Vessey et al., 2008), it would be interesting to expose these two models to complex tasks as described in our paper and determine if similar cognitive deficits could be observed.

In conclusion, we presently describe altered social behavior, impaired spatial learning and memory, and defective hippocampal LTP in *Nxf7-*deficient mice, which relate to behavioral and cognitive defects observed in NXF5-deficient humans. Nxf7-deficient mice appear to be a valid model of human NXF5 deficiency. Our data indeed support the hypothesis that mouse Nxf7 is the functional homolog of NXF5, and open the door for detailed molecular and behavioral analyses of the mechanistic basis of Nxf7/NXF5-dependent cognitive dysfunction.

## Conflict of Interest Statement

The authors declare that the research was conducted in the absence of any commercial or financial relationships that could be construed as a potential conflict of interest.
